# Virtual Reality Human–Human Interface to Deliver Psychotherapy to People Experiencing Auditory Verbal Hallucinations: Development and Usability Study

**DOI:** 10.2196/26820

**Published:** 2021-06-01

**Authors:** Mischa Brander, Stephan T Egger, Noa Hürlimann, Erich Seifritz, Robert W Sumner, Stefan Vetter, Stéphane Magnenat

**Affiliations:** 1 Game Technology Center Department of Computer Science Eidgenössische Technische Hochschule Zürich Zurich Switzerland; 2 Psychotherapy and Psychosomatics Department of Psychiatry Psychiatric University Hospital of Zurich, University of Zürich Zurich Switzerland; 3 Department of Psychiatry Faculty of Medicine University of Oviedo Oviedo Spain

**Keywords:** system usability, virtual reality psychotherapy, verbal auditory hallucinations

## Abstract

**Background:**

Digital technologies have expanded the options for delivering psychotherapy, permitting for example, the treatment of schizophrenia using Avatar Therapy. Despite its considerable potential, this treatment method has not been widely disseminated. As a result, its operability and functionality remain largely unknown.

**Objective:**

We aimed to study the usability of a therapeutic virtual reality human–human interface, created in a game engine.

**Methods:**

Participants were psychiatric hospital staff who were introduced to the therapeutic platform in a hands-on session. The System Usability Scale (SUS) was employed for evaluation purposes. Statistical evaluation was conducted using descriptive statistics, the chi-square test, analysis of variance, and multilevel factor analysis.

**Results:**

In total, 109 staff members were introduced to the therapeutic tool and completed the SUS. The mean SUS global score was 81.49 (SD 11.1). Psychotherapists (mean 86.44, SD 8.79) scored significantly higher (*F*_2,106_=6.136; *P*=.003) than nursing staff (mean 79.01, SD 13.30) and administrative personnel (mean 77.98, SD 10.72). A multilevel factor analysis demonstrates a different factor structure for each profession.

**Conclusions:**

In all professional groups in this study, the usability of a digital psychotherapeutic tool developed using a game engine achieved the benchmark for an excellent system, scoring highest among the professional target group (psychotherapists). The usability of the system seems, to some extent, to be dependent on the professional background of the user. It is possible to create and customize novel psychotherapeutic approaches with gaming technologies and platforms.

**Trial Registration:**

Clinicaltrials.gov NCT04099940; http://clinicaltrials.gov/ct2/show/NCT04099940

## Introduction

Psychotherapy is an effective and cost-efficient method for the treatment of psychiatric and psychological disorders [1]. Over the last few decades, it has been evolving continuously, demonstrating both feasibility and efficacy in practically all diagnostic categories. Indeed, in several categories, it has become the first line of treatment [2,3]. In patients with schizophrenia, psychotherapy as a treatment option has been largely neglected. Recently, however, it has gained recognition as an effective treatment when used in conjunction with pharmacotherapy [4,5]. Furthermore, current guidelines now recommend the early implementation of psychotherapy in the treatment process [6,7].

Psychotherapeutic treatment using digital technologies, virtual reality in particular, has been shown to be at least as efficacious as other treatments [8]. In some fields, particularly schizophrenia, digital technologies have considerably extended therapeutic options [5,9,10], for example, with the novel implementation of Avatar Therapy, whereby psychotherapy is delivered through a computer interface [11,12]. Patients with auditory verbal hallucinations create an avatar of a human entity, to which they attribute the voices. With the help of a therapist, they progressively gain control over the voices, which leads to a reduction of symptoms and distress while increasing quality of life [12].

Despite encouraging early studies and its vast potential, psychotherapeutic treatment using digital technologies has still not been widely disseminated in research or clinical practice [10]. We attribute the limited deployment partially to unavailability as off the shelf tools, making implementation difficult [13,14]. From previous research, it is known that for the optimal delivery of therapy through digital technologies, besides availability, the operability and functionality of the technology are crucial; only once these are well established can the therapist confidently utilize digital technology [14,15]. Moreover, the proper use of such technology is essential for the optimal delivery of the therapy, thus allowing the therapist to develop their therapeutic skills [10].

In this paper, we present a human–human interface that we developed for use in the treatment of patients experiencing verbal acoustic hallucinations. As the usability of the system is a prerequisite for its clinical application, we systematically sought input from nonpatient users [16]. Within mixed skill-grade users, we sought to determine what influence professional background and therapeutic skills have in relation to the use of the therapeutic system.

## Methods

### Virtual Reality Human–Human Interface

Building upon previous studies [11,17,18], we have created a virtual reality human–human interface using the Unity game engine (Unity Technologies) to deliver Avatar Therapy for people experiencing auditory verbal hallucinations. The basic design employs 2 separate apps running on different devices connected via a network, including bidirectional audio (full-duplex voice over internet protocol connection) communication. The first computer hosts a personal avatar creation tool (Virtual Reality Avatar-Creation Tool, VRAT-CT) to design and customize a humanoid avatar, to which patients attribute their auditory verbal hallucinations. This computer also renders the virtual reality through a head-mounted display for the therapeutic session. The voice of the therapist is modulated through a voice transformer (Roland VT-4) to match the auditory verbal hallucination. The therapeutic session is initialized and controlled from the second computer with a Control Center (VRAT-CC) which allows the therapist to control the Avatar and to speak through the Avatar using lip synchronization.

### Software and Hardware

The Unity game engine is a freely available platform for game development. Over 60% of current virtual and augmented reality content have been created with Unity [19]. It provides a 3D editor, a scripting application programming interface written in C# which allows all components to be brought together, and supports 3D graphics, socket communication, and virtual reality (VR). Both apps are created with Unity (version 2019.3.7) and the associated scripts are written in C# with Microsoft Visual Studio 2017, an integrated development environment. For the 3D character, the Multipurpose Avatar package (version 2.11.5; Unity Technologies) was used. To increase the impression that the avatar is speaking, the SALSA LipSync Suite package (version 2.5.0.92; Crazy Minnow Studio LLC) is used to synchronize lip and mouth movements to the voice input of the therapist.

**Figure 1:**
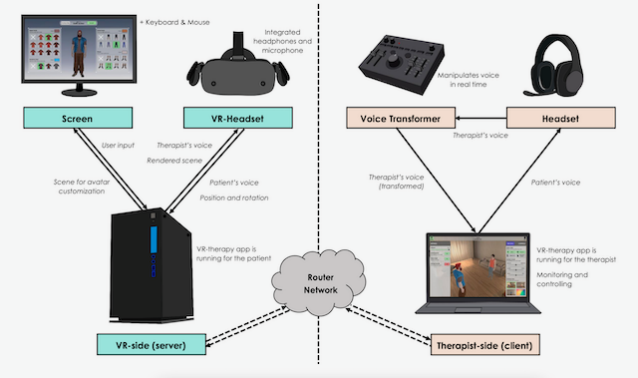
Virtual reality (VR) human–human interface.

The Reverb (Hewlett Packard) headset is used as a head-mounted display. The headset provides a resolution of 2160×2160 per eye at 90 Hz and with a 114° field of view; however, this comes with a series of minimum computational requirements. The producer recommends, at minimum, a Nvidia GeForce GTX 1080 graphic card or an AMD Radeon Pro WX 8200, an Intel Core i7 processor, and 16 GB of RAM. For the operating system, Windows 10 (version 1809 or later) is needed. For this project, a Roland VT-4 voice transformer was used, which provided a set of options for manipulating a voice in real-time. Pitch and format frequency, which can be set with sliders for a deeper or higher voice, were relevant for creating the avatar voice. Figure 1 shows an overview of the software and hardware setup (Multimedia Appendix 1).

### System Usability Scale

The System Usability Scale (SUS) is a tool for measuring the usability of a wide variety of products and services including hardware, software, mobile devices, websites, and apps [18,20]. It is a 10-item questionnaire, with a 5-point Likert Scale from 1 (strongly disagree) to 5 (strongly agree) (Textbox 1). Scale items alternate between positive and negative statements; therefore, correction is required for scoring. For odd-numbered items, the value 1 is subtracted from the user's response whereas for even-numbered items, the user's response is subtracted from 5, yielding a score from 0 to 4 for each item. For interpretation, scores are summed and multiplied by a factor of 2.5. The final score ranges from 0 to 100 [20].

**System Usability Scale.**

1. I think that I would like to use this system frequently.
2. I found the system unnecessarily complex.
3. I thought the system was easy to use.
4. I think that I would need the support of a technical person to be able to use this system.
5. I found the various functions in this system were well integrated.
6. I thought there was too much inconsistency in this system.
7. I would imagine that most people would learn to use this system very quickly.
8. I found the system very cumbersome to use.
9. I felt very confident using the system.
10. I needed to learn a lot of things before I could get going with this system.

### Participants and Assessment

Employees (irrespective of professional background and occupation) of the Psychiatric University Hospital of Zürich were invited to view and test the virtual reality human–human interface used to deliver Avatar Therapy to people experiencing auditory verbal hallucinations. Basic demographic characteristics (age, gender, and occupation) were gathered. Participants were divided into 3 categories: psychotherapists (either psychiatrists or psychologists), nursing staff, and administrative personnel.

### Procedure

Participants were individually informed about the nature of the study and introduced to the therapeutic platform in a hands-on session. They were provided with information about the theoretical background of the therapy and the design and implementation process of the virtual reality human–human interface in practice. Each step of the therapeutic process was explained. Afterward, they created an avatar and customized its voice before experiencing it through VR. Thus, they first created and customized their Avatar for therapy in the patient’s role, after which they carried out a session in the therapeutic role. After the session, participants completed the SUS for each component.

### Statistical Analysis

Descriptive statistics (percentages, means, standard deviations) were used to represent the demographic characteristics of the sample. Differences in the sample were calculated using the chi-square test for proportions. An analysis of variance was performed on continuous variables. The SUS score for the system was calculated. Scores for the avatar creation tool (VRAT-CT) and the control center (VRAT-CC) were evaluated separately. The SUS was evaluated at both item level and global level.

Additionally, a multilevel factor analysis was conducted. Statistical analysis was performed using the statistical language program (version 4.0.3, The R Project).

### Ethics

The study was designed to comply with current ethical standards and local regulations. The ethics committee of the Canton of Zürich approved the study protocol (BASEC 2019-01386). The study was registered at Clinicaltrials.gov (NCT04099940).

## Results

### Sample Demographics

In total, 109 staff members were introduced to the therapy. The sample comprised psychotherapists (n=40), nursing staff (n=43), and administrative personnel (n=26); with a mean age of 34.76 (SD 12.69); 74 participants were female (67.9%). There were no statistically significant differences regarding age or gender distribution among the different professions (Table 1).

**Table 1:** Sample characteristics and outcome evaluation.

Results			Profession							Statistics		
		Psychotherapists (n=40)		Nursing staff (n=43)		Administrative personnel (n=26)		*F* test (*df1,df2*)			*P* value	
Age, mean (SD)			33.25 (9.00)		33.51 (15.85)		30.15 (10.60)		2.09 (2, 106)			.13
**Gender, n (%)**									4.451 (2, 109)^a^			.11
	Male	19 (47)		12 (28)		7 (27)						
	Female	21 (53)		31 (72)		19 (73)						
**SUS^b^ score, mean (SD)**												
	Global	86.44 (8.79)^c^		79.01 (13.30)		77.98 (10.72)		6.136 (2, 106)			.003	
	Virtual reality avatar	87.00 (9.83)^c^		79.71 (13.56)		78.08 (12.50)		5.597 (2, 106)			.005	
	Control center	85.88 (9.53)^c^		78.31 (14.54)		77.88 (11.68)		5.064 (2, 106)			.008	

^a^Chi-square test statistic (*df1*,*df2*).

^b^SUS: System Usability Scale.

^c^Posthoc analysis with Bonferroni correction: psychotherapists scores were greater than nursing staff scores and administrative personnel scores.

### Evaluation Outcomes, System Usability Scale

**Figure 2:**
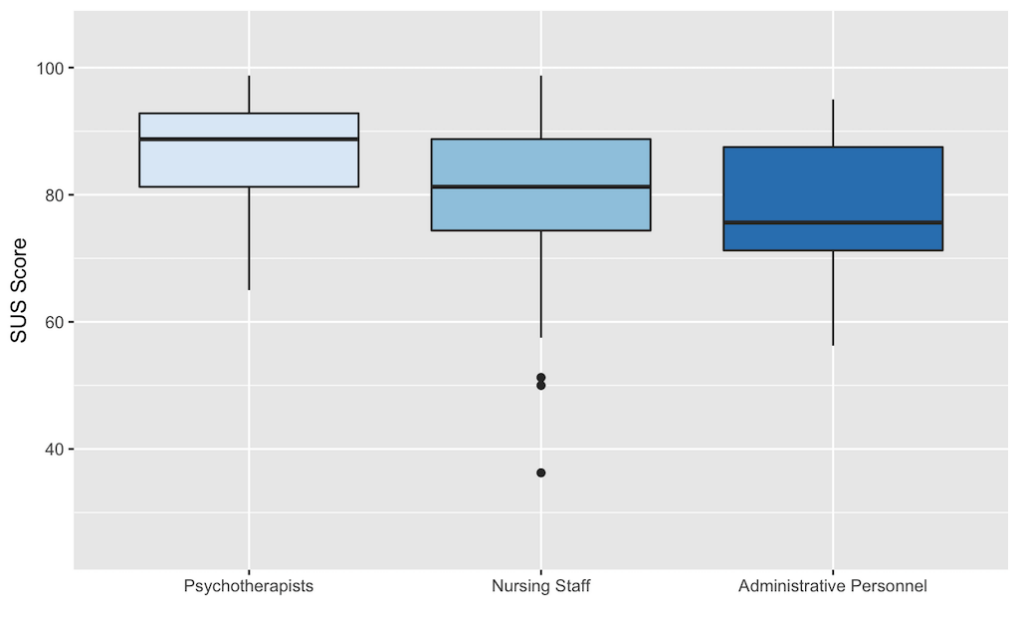
System Usability Scale (SUS) scores. Psychotherapists scored significantly higher than nursing staff and administrative personnel (*P*=.003).

There were no missing items; therefore, no imputation of values was necessary. The SUS scores were normally distributed with few outliers. The mean SUS global score was 81.49 (SD 11.10). The mean score for the VRAT-CT was 82.00 (SD 12.55), and the mean for the VRAT-CC 80.99 (SD 12.67). Male participants scored slightly higher (mean 81.71, SD 15.24) than female participants (mean 81.39, SD 11.19), but this difference was not statistically significant (*P*=.11). Among the professional groups, psychotherapists (mean 86.44, SD 8.79) scored higher than nursing staff (mean 79.01, SD 13.30) and administrative personnel (mean 77.98, SD 10.72). The difference between psychotherapists and other professional groups reached statistical significance (*F*_2,106_=6.136; *P=*.003) (Table 1 and Figure 2).

### Multilevel Factorial Analysis

The System Usability Scale produced a Cronbach α=.80 with good correlation between single items. Item loadings ranged from 0.2 to 0.8 (chi-square *P*<.001; comparative fit index 0.905; sample-size adjusted Bayesian information criterion 5028.08; root mean square error of approximation 0.075) and demonstrated different factor structures for each profession (Table 2).

**Table 2:** Mean score on the System Usability Scale and loadings for each item.

Item	Profession									Statistics		
	Psychotherapists			Nursing staff			Administrative personnel					
	Loadings	Mean (SD)	Loadings		Mean (SD)	Loadings		Mean (SD)	*F* test (*df1,df2*)		*P* value	
1	0.489	4.42 (0.67)^b^	0.356		4.16 (0.79)	0.396		3.92 (1.04)	6.048 (2, 215)		.003	
2	0.591	1.25 (0.46)^b^	0.670		1.35 (0.59)^c^	0.687		1.60 (0.66)	5.993 (2, 215)		.003	
3	0.672	4.45 (0.57)	0.665		4.28 (1.01)	0.565		4.33 (0.86)	1.929 (2, 205)		.15	
4	0.520	2.00 (1.07)^a,b^	0.428		2.49 (1.26)	0.328		2.81 (1.28)	97.663 (2, 215)		<.001	
5	0.500	4.44 (0.65)	0.405		4.31 (0.58)	0.575		4.38 (0.53)	0.896 (2, 215)		.41	
6	0.523	1.30 (0.54)^a,b^	0.513		1.73 (0.90)	0.579		1.79 (0.64)	10.070 (2, 215)		<.001	
7	0.335	4.31 (0.76)	0.805		4.24 (0.85)	0.554		4.08 (0.62)	1.351 (2, 215)		.22	
8	0.501	1.30 (0.58)^a^	0.506		1.71 (1.13)	0.793		1.40 (0.57)	4.946 (2, 215)		.008	
9	0.561	4.22 (0.73)^b^	0.523		3.90 (1.01)	0.5552		3.81 (0.97)	4.219 (2, 215)		.02	
10	0.291	1.50 (0.78)^b^	0.566		2.02 (1.13)^c^	0.535		1.73 (0.79)	6.519 (2, 215)		.002	

^a^Posthoc analysis with Bonferroni correction: psychotherapists scores were greater than nursing staff scores and administrative personnel scores.

^b^Posthoc analysis with Bonferroni correction: psychotherapists scores were greater than administrative personnel scores.

^c^Posthoc analysis with Bonferroni correction: nursing staff scores were greater than administrative personnel scores.

## Discussion

The usability of a digital psychotherapeutic tool developed using a game engine was studied in different professions. The SUS score obtained for the virtual reality human–human interface achieved the benchmark for an excellent system [21,22], scoring highest among the professional target group. The sample's demographic characteristics did not affect these results: the SUS scores were similar regardless of age or gender. To the best of our knowledge, this is the first study to assess the usability of a psychotherapeutic VR treatment tool for people experiencing acoustic verbal hallucinations.

Our study's main strengths are the large sample size and the naturalistic design, particularly the personalized introduction and the practical hands-on approach to the system [14,15]. We chose this approach to emulate the introduction and instruction used by psychotherapists in research and clinical practice. Furthermore, through the personalized introduction to the system, we sought to compensate for differences in the theoretical backgrounds of the professional groups.

The SUS was originally developed to evaluate the usability of products and services, including hardware, software, mobile devices, websites, and apps. Because the product was a combination of these elements and has previously been used to test medical devices and products [23-25], we selected the SUS to enable easy comparison with both similar and dissimilar products or devices [21]. The digital therapeutic system tested yielded a score ranging from good to excellent, depending on professional background [21,22].

The virtual reality human–human interface achieved a higher score among professionals with psychotherapeutic backgrounds (ie, psychiatrists and psychologists). Since all participants were naïve to the system, differences cannot be attributed to user experience [26]. In our opinion, these differences underscore the need for relevant training and professional background in order to fully understand and use the virtual reality therapeutic tool that we have created [14]. Posthoc analysis revealed no differences between psychiatrists and psychologists. We, therefore, consider both to form a uniform group with psychotherapeutic training as the common factor (in Switzerland) [27-29]. In addition, the similarities between psychologists and psychiatrists, regarding educational level and awareness of relevant research, should also be taken into account [30].

The SUS scale was designed as a global measure of perceived usability. Attempts thus far to identify an underlying factor analysis have been misleading and mainly reflected its alternating structure [31]. Nonetheless, we analyzed the SUS at an item level to discern differences potentially attributable to the skill-grade mix of the participants. The SUS scale yielded similar scores between psychotherapists and nonpsychotherapists only for items 3, 5, and 7. These items are more closely related to the handling of the system than to its actual implementation and use in research and clinical practice. The SUS scale also has a different factor structure for each profession, indicating different evaluation patterns for the usability of the system. This leads us to believe that the system is generally easy to use, allowing therapists to quickly become familiar with it and develop confidence, thereby increasing the likelihood of incorporating this system into their therapeutic repertoire and using it to deliver therapy [15,32].

Our study has several limitations that must be acknowledged. First, we did not include a clinical population. Although people experiencing verbal acoustic hallucinations were involved in the development process [16], they were not systematically involved in evaluating the system. At this stage, we chose to focus on the therapeutic end user since they would be responsible for introducing and guiding patients through the system and conducting the therapy sessions afterward. Another factor in our study was the use of only a single session for evaluation. This approach was chosen with the intention of assessing the intuitive usability of the system and to avoid learning effects. We did not compare our therapeutic system with those based on other technical possibilities, such as a non-VR presentation of the Avatar or the use of mobile or handheld devices. It is possible that such technical alternatives may yield a higher usability score. Finally, although no discomfort or side effects were reported, we did not systematically assess these important issues related to the use of VR technology [16].

We were able to demonstrate that a virtual reality human–human interface for research and clinical practice can be developed using an existing and widely available game engine. The results show that the usability of the digital therapeutic tools depends not only on the system itself but also on the user's professional background. We believe this system may enable and encourage psychotherapists to expand their therapeutic skills, to routinely using this technology in research and clinical practice [13,33-35]. In summary, given the high usability scores, gaming technology and platforms seem to be suitable for the creation and customization of novel therapeutic approaches in psychiatry.

## Appendix

Multimedia Appendix 1 Virtual reality human–human interface.

## Abbreviations

SUS: System Usability Scale
VR: virtual reality
VRAT: Virtual Reality Avatar-Creation Tool
